# Calibration of Scintillation Cells for Radon-222 Measurements at the U.S. Environmental Protection Agency

**DOI:** 10.6028/jres.095.016

**Published:** 1990

**Authors:** E. L. Sensintaffar, S. T. Windham

**Affiliations:** U.S. Environmental Protection Agency, Montgomery, AL 36109

**Keywords:** calibration, radon measurement, scintillation cell, uncertainty

## Abstract

Zinc sulfide coated scintillation cells are the primary method for measuring radon-222 at the U.S. Environmental Protection Agency (EPA), Office of Radiation Programs (ORP), Eastern Environmental Radiation Facility (EERF). These cells are used to measure concentrations of radon in exposure chambers that are used to calibrate or test other devices or instruments. Individual cells are calibrated by analyzing samples of air with known concentrations of radon produced by emanation of radon from standard radium-226 solutions obtained from the National Institute of Standards and Technology. The calibration procedure includes ingrowth of radon-222 into equilibrium with the radium in the standard solution, transfer from the solution into an evacuated container, and dilution with a measured volume of air. Samples of the radon in air mixture are transferred to evacuated scintillation cells and sealed for 4 h prior to counting, which allows secular equilibrium to be established between the radon and its decay products.

Calibration factors for each individual cell are computed by decay correcting the radon to the time of collection and calculating the ratio of count rate (cpm), corrected for background, to radon activity (Bq) for the specific volume of the cell. Four or more calibration factors are determined for each cell and aver-aged to provide the calibration factor used for measurements. Calibrations are repeated at 6-mo intervals, and the results of each calibration are compared to the previous averages. When calibration factors fall outside the 95% confidence interval, they are rejected and the cell is checked for defects prior to recalibration.

## 1. Introduction

**Figure 1:**
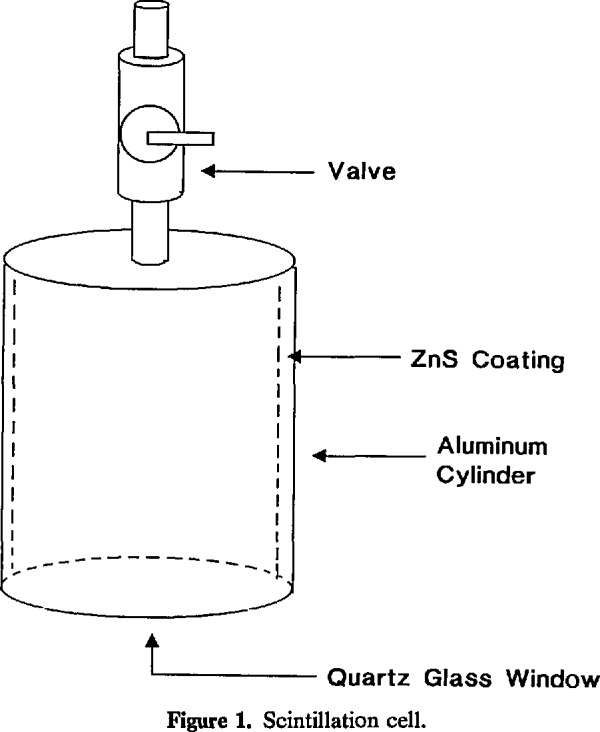
Scintillation cell.

The Environmental Protection Agency (EPA) recently has recognized radon-222 in indoor air as a significant threat to the health of the public, which has resulted in very rapid growth of the radon measurement industry in the United States. The EPA has initiated programs, such as the Radon Measurement Proficiency Program and individual testing and exposure programs, to help other federal and state agencies assure accurate measurements. The principal measuring device used by EPA to establish radon concentrations for these tests is the scintillation cell. Measurements made with commercially available scintillation cells with a volume of 0.125 × 10^−3^ m^3^ (see fig. 1) are used as the basis for determining radon concentrations in two radon exposure chambers. These chambers are used to calibrate and test other measurement devices, so it is very important that they be accurate. Consequently, the cells must be accurately calibrated with reliable sources of radon-222.

## 2. Calibration

**Figure 2:**
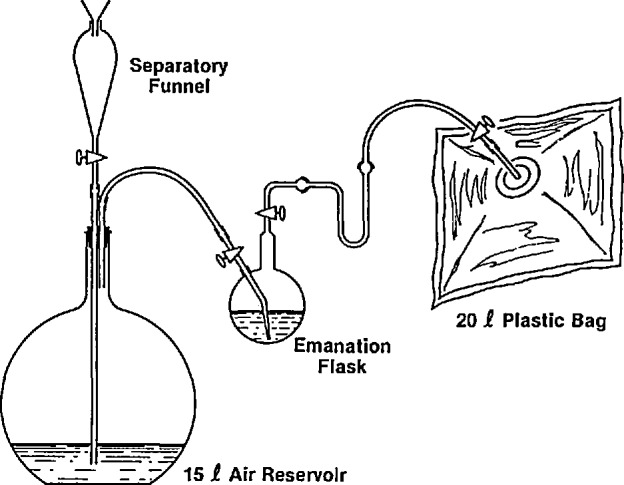
Radon emanation system.

Scintillation cells are calibrated by filling evacuated cells with radon that has emanated from a radium-226 solution obtained from the National Institute of Standards and Technology. The procedure used, which has been described by Lucas [1], employs water displacement to force a measured amount of air through an emanation flask that contains the standard radium solution (see fig. 2). The 5-10 L of air required to purge the radon from the flask flows into a previously evacuated plastic bag. Although the bag has a usable volume of approximately 0.02 m^3^, when the input is limited to 0.01 m^3^ or less, the pressure inside the bag is negUgible and no correction for pressure is required. The radon concentration is determined by dividing the activity of radon in becquerels (Bq) emanated from the radium solution by the volume of air in cubic meters (m^3^) transferred to the bag.

**Figure 3:**
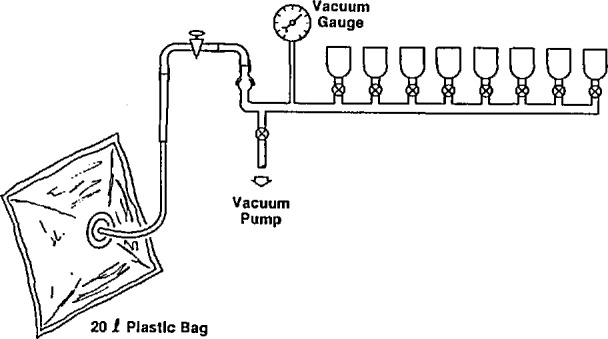
Radon transfer manifold.

Scintillation cells are filled with the radon and air mixture through a manifold and vacuum pump assembly as shown in figure 3. The cells and manifold are evacuated with a vacuum pump and then filled with the radon mixture from the bag. This technique allows several cells to be filled from a single emanation procedure. After filling, the cells are set aside for at least 4 h to allow ingrowth of radon decay products. The cells are analyzed for radon content by counting light pulses on a 2-in photomultiplier for 400 min.

Calibration factors are calculated for each cell using eq (1):

$$CF=\frac{\left(R-B\right)}{Rn}\cdot \frac{C}{A},$$ (1)

where

*CF* = Calibration factor (cpm/Bq),
*R* = Total count rate (cpm),
*B* = Background count rate (cpm),
*Rn* = Radon-222 concentration (Bq m^−3^),
*V* = Volume of cell (m^3^),
*C* = Correction for decay during counting,

$$C=\frac{\lambda t}{1-e^{-\lambda t}},$$

*A* = Correction for decay prior to counting.
*A* = e^−λ^*^T^*,
λ = decay constant for radon-222 (min^−1^),
*t* = counting time (min), and
*T* = elapsed time between collection of sample and counting (min).

A typical value for calibration factors for our 0.125×10^−3^ m^3^ scintillation cells is 135 cpm/Bq. Calibrations are repeated a minimum of four times and the average calibration factors are calculated. Typical data for 10 scintillation cells are shown in table 1. Calibrations are also repeated periodically and checked against previous data for indications of cell damage or aging. If the calculated calibration factor falls within the 95% confidence interval for that cell, it is added to the data base for that cell. If the factor falls outside the 95% confidence interval, the cell is inspected for damage or leakage and repaired if necessary.

## 3. Uncertainty of the Calibration

The relative systematic standard deviation for a single determination when using this method is estimated, by Lucas [1], to be 1.4%. The total uncertainty associated with a single radon measurement using our 0.125×10^−3^ m^3^ scintillation cells calibrated with this method is described by Pohl [2] with the following relationship:

$$\%U={\left[\left(G+b)/{\left(G-b\right)}^{2}+{\left(0.0175\right)}^{2}\times (1/J\right)\right]}^{1/2}\times 100$$ (2)

where

% *U* = Total uncertainty expressed as a percent,
*G* = Gross counts in time *t*,
*b* = Background counts in time *t*, and
*J* = Number of calibrations for the scintillation cell.

Ar 148 Bq m^−3^ (4 pCi/L) with counting times of 400 min, typical values would be

$$\begin{array}{l} G=1200,\\ b=200,\text{ and}\\ J=4. \end{array}$$

Using eq (2),

$$\begin{array}{l} \%U=[\left(1200+200\right)/{\left(1200-200\right)}^{2}+{\left(0.0175\right)}^{2}\\ \;\times 1/4){]}^{1/2}\times 100\\ \%U=3.8\%\left(1\;\text{sigma}\right) \end{array}$$

## 4. Conclusions

This procedure has proven to be an easy and reliable method that allows calibration of multiple scintillation cells for radon-222 measurements. Intercomparisons have shown that EERF radon-222 measurements are comparable to those obtained by other laboratories.

## Figures and Tables

**Table 1:** Example cell calibration factors

Cell No.	*CF*_1_	*CF*_2_	*CF*_3_	*CF*_4_	$\bar{CF}$	SD	SD/*CF*(%)
4	135	148	139	143	141	6	3.9%
9	122	138	130	120	128	8	6.4%
10	140	144	142	146	143	3	1.9%
14	133	142	130	139	136	6	4.0%
18	145	143	141	142	143	1	1.2%
25	143	145	139	148	144	4	2.6%
31	139	149	141	139	142	5	3.4%
41	131	141	134	139	136	4	3.4%
43	133	144	136	143	139	5	3.9%
44	136	144	142	145	142	4	2.8%
